# High mobility group box 1 contributes to wound healing induced by inhibition of dipeptidylpeptidase 4 in cultured keratinocytes

**DOI:** 10.3389/fphar.2015.00126

**Published:** 2015-06-16

**Authors:** Tiziana Sinagra, Sara Merlo, Simona F. Spampinato, Rocco De Pasquale, Maria Angela Sortino

**Affiliations:** ^1^Section of Pharmacology, Department of Biomedical and Biotechnological Sciences, University of Catania, Catania, Italy; ^2^Department of General Surgery and Medical-Surgical Specialties, University of Catania, Catania, Italy

**Keywords:** skin, diabetes, keratinocytes, HMGB1

## Abstract

Dipeptidyl peptidase 4 (DPP4) is expressed in various tissues, including the skin, and DPP4 inhibitors, that are currently used for the treatment of diabetes, may be effective also for complications of diabetes that affect the skin. To assess the role of DPP4 in keratinocytes, after creating a scratch wound in a monolayer of NTCC 2544 cells, we evaluated DPP4 expression and monitored wound repair over time, after treatment with the DPP4 inhibitor 1(((1-(hydroxymethyl)cyclopentyl)amino)acetyl)2,5-cis-pyrrolidinedicarbonitrile (DPP4-In). Expression of DPP4 increased early and was maintained up to 48 h following the scratch as shown by western blot and immunostaining. Treatment with 10 μM DPP4-In reduced DPP4 expression and significantly accelerated wound repair. This effect did not involve enhanced cell proliferation as shown by MTT proliferation assay, the lack of changes of cell cycle profiles and the slight inhibition of ERK phosphorylation. Enhancement of wound repair by DPP4 inhibition was prevented by the non-specific MMPs inhibitor GM6100 (5 μM). Treatment with DPP4-In increased the expression of high mobility group box 1 (HMGB1), a substrate of this enzyme, and exposure of NCTC 2544 cells to DPP4-In and exogenous HMGB1 (10 nM) produced a non-additive effect. Finally the healing promoting effect of DPP4-In was prevented by pretreatment with a neutralizing anti-HMGB1 antibody. The present results suggest that DPP4 inhibition contributes to enhanced wound healing by inducing keratinocytes to migrate into a scratched area. This effect seems to be independent of cell proliferation and involves enhanced production of HMGB1.

## Introduction

CD26, a 110 kDa cell surface glycoprotein, is expressed in several cell types and is endowed with dipeptidyl peptidase 4 (DPP4) activity in its extracellular domain (Ulmer et al., 1990; Ohnuma et al., 2008). It acts as a serine peptidase that cleaves N-terminal dipeptides when proline, hydroxyproline and alanine are present at the penultimate position (De Meester et al., 1992). In various cell types CD26/DPP4 exerts different properties and, among others, it is involved in cell proliferation, signal transduction, cytokine production and T cell activation (Hegen et al., 1997; Reinhold et al., 1998). In the last several years the interest toward DPP4 enzymatic activity has greatly increased due to the ability of DPP4 to cleave the incretin hormone, glucagon-like peptide-1 (GLP-1), an insulin secretagogue (Deacon et al., 1995; Pauly et al., 1996). Inhibitors of DPP4 have been then developed and are widely used as effective agents for the treatment of type 2 diabetes mellitus (DMT2; Pratley and Salsali, 2007). Interestingly, the effects of GLP-1 and GLP-1 analogs, as well as of DPP4 inhibitors, that act by inhibiting the cleavage of endogenous GLP-1, are not limited to the control of hyperglycemia, but involve several other tissues and functions (Sortino et al., 2013; Gallwitz, 2014). Particularly relevant appears the role of DPP4 in the skin (Fuchs et al., 2009). Interest in this regard has been raised due to the frequently observed cutaneous complications occurring during diabetes (Quondamatteo, 2014) and the ensuing potential of DPP4 inhibitors to control them. Among cutaneous complications, one of the most common, i.e., the reduced healing capacity of diabetic skin (Falanga, 2005), seems to directly involve DPP4. The kinetics of DPP4 expression after wound differs in fact in control and *ob/ob* diabetic mice, and the reappearance of DPP4, after an initial reduction of its expression, coincides with a resolved wound condition in healthy animals, but with the persistence of an inflammatory status, that impairs wound repair, in diabetic mice (Schurmann et al., 2012). More importantly, inhibition of the enzymatic activity results in enhanced re-epithelialization in impaired wound healing in diabetic animals (Schurmann et al., 2012). Improvement of wound repair by the DPP4 inhibitor linagliptin has been related to increased levels of GLP-1 in the wound area that would reduce the inflammatory reaction impairing the re-epithelialization process. However, factors that contribute to the reparative process in the skin are complex and different cell types are involved. Very little data exist regarding the role of DPP4 specifically in keratinocytes although this cell type represents one of the major sources of the enzyme in the skin and plays a crucial role in the reparative process. In the present study attention has then been focused specifically on keratinocytes in order to analyze the function of DPP4 and the effect of its inhibition in an *in vitro* model of wound repair.

## Materials and Methods

### Drugs and Reagents

The DPP4 IV inhibitor III, 1(((1-(hydroxymethyl)cyclope-ntyl)amino)acetyl)2,5-cis-pyrrolidinedicarbonitrile (DPP4-In) (Merck Millipore, Darmstadt, Germany) was dissolved in 100% ethanol at an initial concentration of 100 mM and all subsequent dilutions were made in water. Chemotaxis-HMGB1, LPS-free (HMGBiotech, Milan, Italy) was dissolved in water and recombinant human SDF-1α (Peprotech, Rocky Hill, USA) was prepared in a 0.1% BSA solution. In-solution GM6001, was from Calbiochem® (Merck KGaA, Darmstadt, Germany). Mouse monoclonal anti-HMGB1 was from HMGBiotech and anti-SDF1α and anti-CD26 were provided by Santa Cruz Biotechnology (Santa Cruz, USA). Anti-ERK and –pERK antibodies were from Cell Signaling (Milan, Italy). Secondary antibodies IR680 and IR800 were provided by MMedical (Milan, Italy). All cell culture plastics were from BD Falcon (Milan, Italy) and common cell culture reagents including media, media supplements, serum, trypsin, buffers and antibiotics were from Invitrogen Srl (Milan, Italy). Mouse anti-α-tubulin and all other reagents, unless otherwise specified were from Sigma Aldrich (St Louis, USA).

### Cell Cultures

NCTC 2544 human keratinocyte cells (Interlab Cell Line Collection, Genoa, Italy) were grown at 37°C in an atmosphere of 5% CO_2_ in DMEM supplemented with 10% FCS and penicillin/streptomycin. Adult Normal Human Epidermal Keratinocytes (N-HEK) and Human Adult Epidermal Keratinocytes-Diabetic Type II (D-HEK) were maintained at 37°C in an atmosphere of 5% CO_2_ in KGM-Gold Keratinocyte Growth Medium supplemented with KGM-Gold BulletKit (hydrocortisone 0.1%, transferrin 0.1%, epinephrine 0.05%, gentamicin sulfate/amphotericin-B, 0.1%, bovine pituitary extract, 0.4%, epidermal growth factor human recombinant, 0.1% and insulin 0.1%). Cells, media and supplements were all from Lonza (Basel, Switzerland). The primary culture of skin fibroblasts was obtained from the outgrowth of a skin punch and grown in DMEM supplemented with 10% FCS and penicillin/streptomycin.

### Scratch Wound Assay

NCTC 2544 cells were deprived of serum and scratched with a sterile P200 pipette tip according to a paradigm previously described (Merlo et al., 2009). Serum deprivation was considered necessary to reduce or abolish proliferation that could confound evaluation of the cell migratory process. After removal of the resulting debris by repeated washes, cells were subjected to treatment and scratch wound closure was monitored by phase microscopy capturing images of the same field with a 10X objective at different times, as specified. The cell free area was determined with the aid of the image processing software “Image J” developed by NIH and in public domain.

### Western Blot Analysis

NCTC 2544 cells, maintained in serum-deprived DMEM were subjected to mechanical damage by multiple scratches using a sterile P200 pipette tip. At different times after the induction of the damage (24–96 h), cells were collected and lysed with RIPA buffer, containing a protease and phosphatase inhibitor cocktail mix (Sigma). Full lysis was obtained by sonication. The samples were then centrifuged for 5 min at 4000 rpm to remove insoluble material. Fifty-five to 65 μg of protein extract were separated by SDS-PAGE and transferred to nitrocellulose membranes (Hybond ECL, Amersham Biosciences Europe GmbH) using a transblot semidry transfer cell for 60 min at 0.8 mA/cm^2^. Membranes were then blocked for 30 min in Odyssey Blocking Buffer diluted 1:1 with PBS-Tween20 0.1%, and processed for immunodetection with the following antibodies: rabbit anti-CD26 (1:500), mouse anti-HMGB (1:750), goat anti-SDF-1α (1:200), rabbit anti-ERK and anti-phospho-ERK (1:500), mouse anti-α tubulin (1:6000; Sigma-Aldrich) and IR680 and IR800 secondary antibodies (1:10000). Detection of specific bands was carried out using LI-COR Odyssey scanner.

### Immunocytochemistry

NCTC 2544 cells were fixed in 4% paraformaldehyde, permeabilized with 0.1% Triton X-100 and saturated with 3% BSA. Cells were then incubated with anti-CD26/DPP4 (1: 100) o/n followed by incubation with Alexa 488-conjugated anti-rabbit antibody (1:300) for 60 min at room temperature.

### Cell Cycle Analysis

NCTC 2544 cells were collected with gentle trypsinization, fixed in ice-cold 70% ethanol, treated for 1 h at 37°C with 100 μg/ml ribonuclease to remove RNA. Cells were then incubated with 50 μg/ml propidium iodide just prior to analysis of DNA content and ploidy carried out by a Beckton Dickinson FC500 flow cytometer. Data were processed with the ModFit software for cell cycle analysis.

### Statistics

Data shown are expressed as mean +/– SEM of independent experiments each run in quadruplicates. All data were analyzed by one-way and two-way ANOVA followed by Newman Keuls or Bonferroni *post hoc* test for significance, as indicated. The level of significance was set at *p* < 0.05.

## Results

**FIGURE 1:**
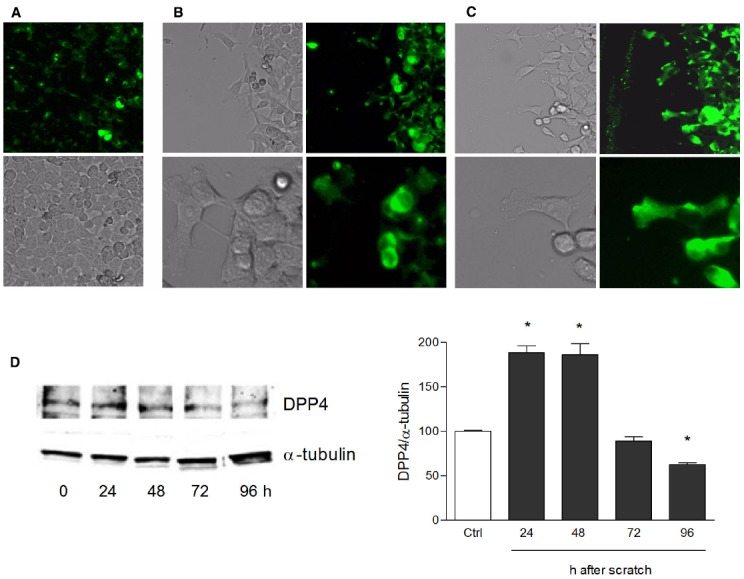
**Increase of DPP4 expression in NCTC 2544 keratinocytes subjected to scratch wound.** DPP4 expression is analyzed under basal conditions **(A)** and following scratch wound **(B,C)**. Expression of the enzyme appears upregulated already 8 h after scratch **(B)** as shown by immunostaining and its overexpression is maintained up to 48 h **(C)**. In **(A)** immunostaining for DPP4 (upper panel) and a bright field image (lower panel) of a NCTC2544 monolayer (10x magnification). In **(B,C)** bright field and immunofluorescence images of the scratched area captured with a 10x (upper panels) and a 40x (lower panels) magnification. Western blot analysis confirms the overexpression at 24 and 48 h **(D)**. Bars show densitometry vs α-tubulin and are mean +/– SE of at least three independent determinations. *p < 0.05 vs control as by one way ANOVA and Newman-Keuls test for significance.

**FIGURE 2:**
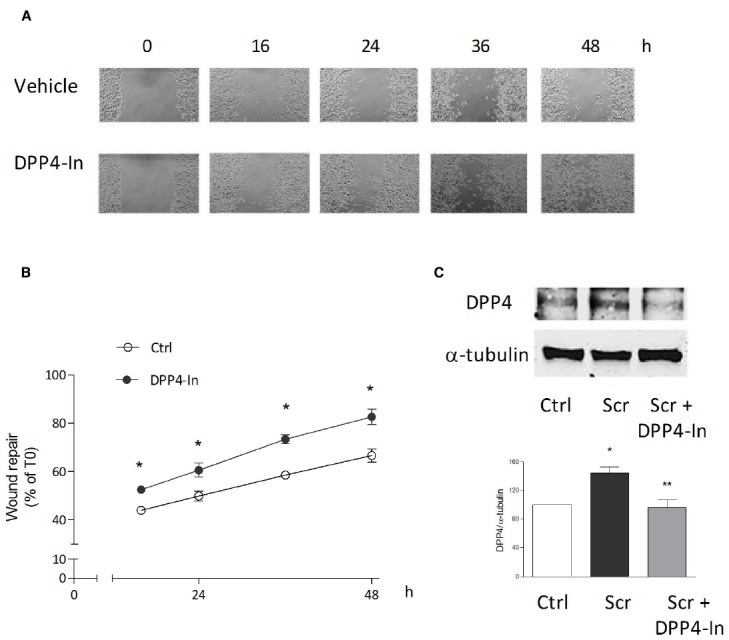
**Inhibition of DPP4 increases wound closure in a monolayer of NTCC 2544 keratinocytes.** Treatment with DPP4-In (10 μM) increases the ability of keratinocytes to migrate into the empty area and to repair the wound at all time points examined **(A,B)**. Representative images of the wound repair over time are reported in **(A)**. This effect is accompanied by reduction of the expression of DPP4 induced by scratch wound (scr), as by western blot analysis **(C)**. Data are mean +/– SE of 3–5 independent experiments each run in quadruplicates. In **(B)**, *p < 0.05 vs control by two-way ANOVA and Bonferroni *post hoc* test for significance; *p < 0.05 vs unscratched and **p < 0.05 vs untreated scratch **(C)** by one-way ANOVA and Newman-Keuls test for significance.

**FIGURE 3:**
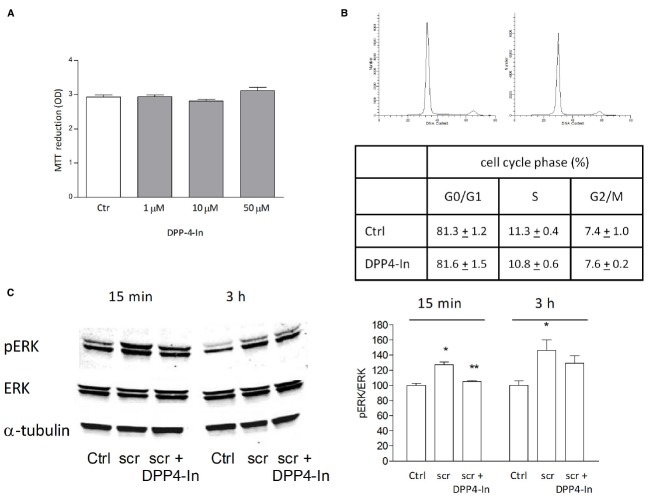
**The healing promoting effect of DPP4-In does not involve increased cell proliferation.** Treatment with increasing concentrations of DPP4-In does not modify cell proliferation as from MTT assay **(A)**. Cell cycle analysis of NCTC 2544 cells treated with DPP4-In (10 μM) for 24 h does not modify the distribution of cells in different phases of cell cycle, as by flow cytometry after labeling of cells with propidium iodide. In **(B)**, representative profiles of control (left panel) and DPP4-In (right panel) treated cells. Data summarized in the table are mean +/– SE of three independent experiments each run in quadruplicates. In **(C)**, the increased phosphorylation of ERK induced by scratch wound is counteracted by DPP4-In (10 μM) treatment at 15 min and at 3 h. One representative plot is shown and expression of α-tubulin is reported for loading control. Data were analyzed by one-way ANOVA and Newman-Keuls test for significance. In **(B)**, *p < 0.05 vs control and **p < 0.05 vs scratch alone.

**FIGURE 4:**
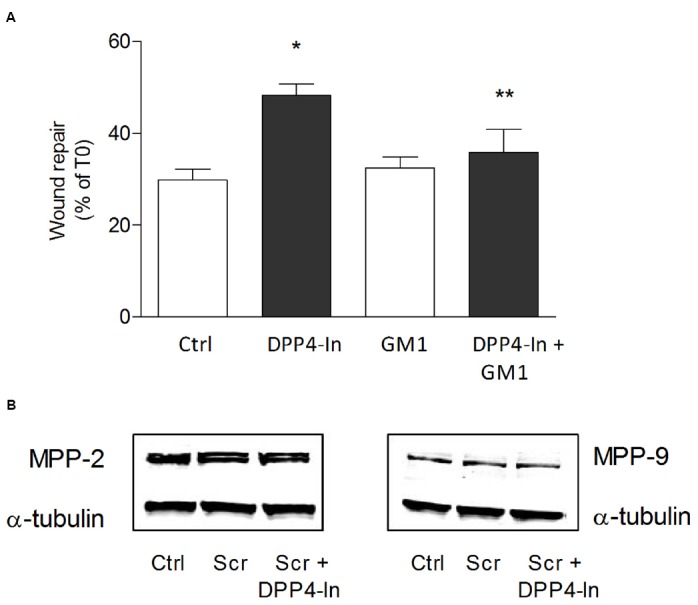
**DPP4-In requires MMPs to promote wound repair.** The cell monolayer was scratched and pre-treated with the non-specific MMP inhibitor GM6001 (5 μM) for 30 min prior to addition of DPP4-In (10 μM) **(A)**. In **(B)** representative blots of MMP2 and MMP9 24 h after scratch in the presence and absence of DPP4-In are shown. Data reported are the % of scratch repair at 24 h and represent mean +/– SE of three independent experiments. *p < 0.05 vs control and **p < 0.05 vs DPP4-In alone by one-way ANOVA and Newman-Keuls test.

**FIGURE 5:**
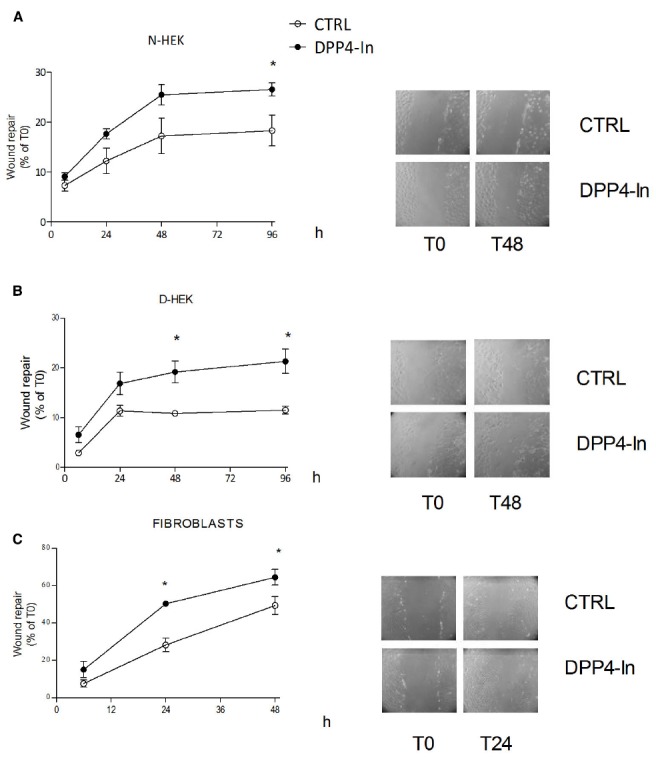
**Inhibition of DPP4 increases scratch repair in different cell types.** Treatment with DPP4-In (10 μM) enhances wound repair in normal human keratinocytes (N-HEK) **(A)**, keratinocytes from diabetic patients (D-HEK) **(B)** and fibroblasts from healthy human skin **(C)**. In **(A)** and **(B)**, DPP4-In was added at time 0 and scratch repair was monitored for the following 96 h. Data are mean +/– SE from three independent experiments each run in quadruplicates. *p < 0.05 by Two-way ANOVA and Bonferroni *post hoc* test for significance.

Human keratinocytes, NCTC 2544 cells, express DPP4 under basal conditions, as detected by immunocytochemistry (Figures 1A–C) and western blot (Figure 1D). When NCTC 2544 cells are subjected to injury by scratching the monolayer to create a wound, the expression of DPP4 increases, especially at the rim of the wound. Immunostaining shows that increased expression is already present at 8 h (Figure 1B) and is maintained 48 h after scratch (Figure 1C). This increase is confirmed by western blot analysis of proteins obtained from a monolayer of NCTC 2544 cells exposed to multiple scratches. Enhanced DPP4 is measurable at 24 h (the earliest time point analyzed) and disappears at 72 h (Figure 1D). NCTC 2544 cells were subjected to a scratch wound migration assay, in which the ability of cells to migrate and cover the empty space is monitored. Untreated cells tend to close the wound by about 60% within 48 h (Figures 2A,B). Treatment with a DPP4 inhibitor (DPP4-In), at the concentration of 10 μM, accelerates wound closure with an effect that appears to be significantly different from control at all time points analyzed (Figure 2B). NCTC 2544 cells exposed to 10 μM DPP4-In for 24 h also show a reduction of DPP4 overexpression induced by scratch injury (Figure 2C). The effect of DPP4-In is exclusively due to stimulation of migration and an effect on cell growth is excluded. Treatment with increasing concentrations of the drug (from 0.1 up to 50 μM) does not affect in fact cell proliferation, as measured by the MTT proliferation assay (Figure 3A), and in parallel does not modify cell cycle profiles, as revealed by flow cytometric analysis of cell cycle (Figure 3B and table). In addition, inhibition of DPP4 decreases (at 15 min), or slightly but not significantly reduces (at 3 h), the enhanced ERK phosphorylation induced by scratch (Figure 3C). Increased migration of keratinocytes induced by DPP4 inhibition seems to involve matrix metalloprotease (MMP) activation. The effect of DPP4-In is in fact prevented when the aspecific inhibitor of MMPs, GM6001, is used. Treatment of a scratched NCTC 2544 culture with 5 μM GM6001, added 30 min before DPP4-In, completely prevents the ability of the drug to accelerate wound closure (Figure 4A). However, no significant increase in the expression of MMP-2 and MMP-9 is detectable following DPP4-In treatment, at least under the experimental conditions that have been used (Figure 4B). The effect of DPP4 inhibition on scratch repair has been analyzed also in N-HEK as well as in human adult epidermal keratinocytes obtained from patients with type II diabetes (D-HEK). Basal expression of DPP4 does not differ between the two cell types, and scratch closure, that proceeds very slowly in both cell types, is significantly improved by chronic exposure to DDP4-In (10 μM) to a similar extent in N-HEK (Figure 5A) and D-HEK (Figure 5B). Only a slight shift to an earlier significant effect of DPP4 inhibition is observed in D-HEK compared to N-HEK cells. In addition, the favoring effect of wound repair is not restricted to keratinocytes, as a better scratch repair is observed also following exposure of human skin fibroblasts to 10 μM DPP4-In (Figure 5C).

**FIGURE 6:**
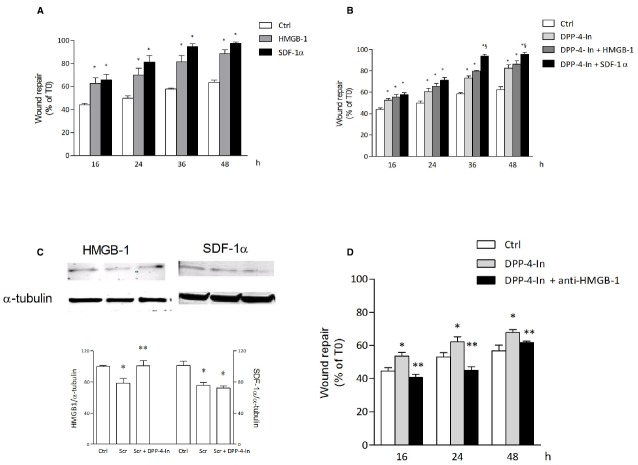
**HMGB1 is involved in the healing promoting effect of DPP4-In.** HMGB1 (10 nM) and SDF-1α (50 ng/ml) significantly increase wound repair over time in scratched NCTC 2544 keratinocytes **(A)**. Co-treatment with DPP4-In (10 μM) and HMGB1 (10 nM) produces a non-additive effect **(B)**, whereas co-addition of DPP4-In and SDF-1α (50 ng/ml) potentiates repair induced by DPP4-In alone **(B)**. Western blot analysis shows that treatment with DPP4-In (10 μM, 24 h) counteracts the reduction of HMGB1, but not that of SDF-1α induced by scratch **(C)**. In **(D)**, treatment with a neutralizing anti-HMGB1 antibody (2.5 μg/ml) prevents the increase of scratch repair induced by treatment with DPP4-In (10 μM). Data are mean +/– SE of three to four independent experiments. In **(A)** and **(B)**, *p < 0.05 vs control; §*p* < 0.05 vs DPP-In alone. In **(C)**, *p < 0.05 vs control and **p < 0.05 vs scratch alone; in **(D)**, *p < 0.05 vs control and **p < 0.05 vs DPP4-In alone. All data were analyzed by one-way ANOVA and Newman-Keuls test for significance.

In search for possible mediators of the inhibition of DPP4 on keratinocyte wound repair, HMGB1 and SDF1α were analyzed. Both these agents are in fact reported to be substrates for DPP4 activity. Treatment of NCTC 2544 cells with 10 nM HMGB1 or 50 ng/ml SDF1α accelerates wound closure with an effect already present at early time points (16 h) and persistent up to 48 h (Figure 6A). Co-treatment of NCTC 2544 cells with DPP4-In (10 μM) and 10 nM HMGB1 produces an effect favoring wound repair superimposable to that obtained with DPP4-In alone (Figure 6B). In contrast, exposure of cells to DPP4-In together with 50 ng/ml SDF1α causes an improved scratch closure, significantly different from that induced by DPP4-In alone (Figure 6B). Importantly, treatment with DPP4-In increases the expression of HMGB1, as detected by western blot analysis (Figure 6C), whereas expression of SDF-1α is not modified. In addition, preincubation with a neutralizing anti-HMGB1 antibody (2.5 μg/ml) prevents the favoring effect of DPP4-In on wound repair (Figure 6D). Conversely, treatment with an anti-SDF1α antibody (5 μg/ml) fails to modify DPP4-In effect (not shown).

## Discussion

Linagliptin, a DPP4 inhibitor used in clinical practice for the treatment of DMT2, has been reported to improve tissue regeneration in diabetic mice (Schurmann et al., 2012). This observation appears particularly relevant due to the impaired wound healing that often occurs as a consequence of diabetes, and the potential use of this class of drugs to control not only hyperglycemia, but also diabetes complications that affect other tissues. Attention then was driven to the expression of DPP4 in injured skin since data obtained in control and diabetic mice reveal that high expression of the enzyme correlates with persistence of an unresolved wound in diabetes (Schurmann et al., 2012). Beside diabetes, the skin may represent a new target organ for DPP4 inhibitors as the enzyme appears upregulated in several skin pathological conditions (reviewed, in Thielitz et al., 2008a).

Major sources of DPP4 in the skin are epidermal keratinocytes and dermal fibroblasts. In the latter cell type, inhibition of DPP4 has been related to reduction of cell proliferation and counteraction of TGF-*β*1-induced effects on collagen production, matrix deposition and fibronectin expression (Thielitz et al., 2008b). We here confirm that DPP4 is expressed also in cultured keratinocytes (Reinhold et al., 1998; Vetter et al., 2000) and report that in NCTC 2544 cells, a well characterized human keratinocyte cell line, expression of DPP4 is increased when a wound is mechanically produced in the monolayer. Inhibition of the activity and expression of DPP4 results in improved wound repair. This effect involves cell migration but not keratinocyte proliferation, as from proliferation assay and cell cycle analysis, and finds support in literature data, where inhibition (Reinhold et al., 1998; Vetter et al., 2000) or no effect (Schurmann et al., 2012) on keratinocyte proliferation by DPP4 inhibition are reported. In addition, the increased ERK phosphorylation induced by the scratch wound is inhibited by treatment with DPP4-In, an effect already observed following DPP4 inhibition in various cell types, including keratinocytes (Thielitz et al., 2008b; Ta et al., 2010; Schurmann et al., 2012).

Interestingly, increased keratinocyte migration and wound closure induced by inhibition of DPP4 activity involves activation of MMPs, as the pharmacological blockade of these enzymes prevents enhanced wound repair. This indirect observation is not supported by increased expression of the two MMPs mainly involved with migration, i.e., MMP2 and MMP9. However, this lack of effect is not completely unexpected as expression of both enzymes is already high and the multiscratch model adopted for quantitative analysis may limit detection of small changes.

An accelerated wound healing by DPP4 inhibition is observed also in primary cultures of keratinocytes from control and diabetic patients. Both these cell cultures proceed very slowly toward wound repair and only a slightly faster healing promoting effect by DPP4-In is observed in cells from diabetic patients. This difference cannot be ascribed to different levels of DPP4 in diabetic skin, according to the comparable expression of DPP4 reported in control and *ob/ob* diabetic mice in intact skin (Schurmann et al., 2012). However, it certainly deserves further attention and other studies are needed to better characterize DPP4 expression and its control in keratinocytes from diabetic skin.

The increase of GLP-1 induced by DPP4 inhibition in the wound area has been indicated as one of the mechanisms involved in the facilitating effect of the healing process (Schurmann et al., 2012). However, keratinocytes are only target of GLP-1 and several other substrates of the enzyme have been described (Mentlein, 1999). Of note, HMGB1 is cleaved and inactivated by DPP4 (Marchetti et al., 2012), is expressed in cultured fibroblasts and keratinocytes (Straino et al., 2008) and DPP4 counteracts HMGB1-induced endothelial cell migration and neovascularization (Marchetti et al., 2012). In line with these observations, we now report that in cultured keratinocytes inhibition of DPP4 causes increased cell migration, very likely through enhanced HMGB1, as suggested by its increased expression following DPP4 inhibition and prevention of DPP4-In-healing promoting effect by treatment with a neutralizing anti-HMGB1 antibody. Accordingly, HMGB1 exerts chemotactic effect on fibroblasts and keratinocytes *in vitro* (Straino et al., 2008), accelerates wound closure in HaCAT cultured keratinocytes (Ranzato et al., 2009) and 3T3 mouse fibroblasts (Ranzato et al., 2010) and improves wound healing in diabetic animals (Straino et al., 2008). Although HMGB1 has been shown to be involved in migratory processes in different cell types (Rauvala and Rouhiainen, 2010), it has been argued that an increase of HMGB1 may be deleterious to cells due to its proinflammatory properties (Schaper and Havekes, 2012). However, we here suggest HMGB1 as a mediator of keratinocyte migration induced by DPP4 inhibition, an effect that may be distinct from its global action in the skin. Furthermore, an increase of HMGB1 following treatment with DPP4 inhibitors has been shown (Marchetti et al., 2012), but the use of this class of antidiabetic drugs in clinical practice has never been associated with appearance of inflammatory effects in various tissues (Schaper and Havekes, 2012).

Finally, our results do not support the possibility that SDF1α, another well described DPP4 substrate, mediates in keratinocytes the improved wound healing induced by DPP4 inhibition. However, despite the major role of SDF1α in wound repair, including diabetic wound healing, these results could be at least partially expected, considering that the major constitutive sources of SDF1α in the skin are not keratinocytes, but endothelial cells and fibroblasts (Avniel et al., 2006; Toksoy et al., 2007).

In conclusion, our results show that inhibition of DPP4 increases the ability of keratinocytes to repair a scratch wound *in vitro*. This effect involves HMGB1 but not SDF1α, two well established substrates of DPP4 endowed with cell migration enhancing properties. The data confirm that DPP4 inhibitors, currently used in clinics for the treatment of DMT2, may be effective also against skin complications that occur during diabetes.

### Conflict of Interest Statement

The authors declare that the research was conducted in the absence of any commercial or financial relationships that could be construed as a potential conflict of interest.
